# Cognitive dysfunction in autoimmune rheumatic diseases

**DOI:** 10.1186/s13075-020-02180-5

**Published:** 2020-04-15

**Authors:** Csaba Oláh, Noa Schwartz, Christopher Denton, Zsófia Kardos, Chaim Putterman, Zoltán Szekanecz

**Affiliations:** 1 Departments of Neurosurgery, Borsod County Teaching Hospital, Miskolc, Hungary; 2 Division of Rheumatology, Albert Einstein College of Medicine, Bronx, NY 10461 USA; 3 Centre for Rheumatology, Royal Free Campus, University College London, London, UK; 4 Departments of Rheumatology, Borsod County Teaching Hospital, Miskolc, Hungary; 5 Department of Microbiology & Immunology, Albert Einstein College of Medicine, Bronx, NY 10461 USA; 6 Division of Rheumatology, Faculty of Medicine, University of Debrecen, Nagyerdei str 98, Debrecen, 4032 Hungary

**Keywords:** Cognitive function, Cognitive dysfunction, Rheumatoid arthritis, Systemic lupus erythematosus, Neuropsychiatric lupus, Systemic sclerosis

## Abstract

For people with chronic autoimmune rheumatic diseases (AIRD), such as rheumatoid arthritis (RA), systemic lupus erythematosus (SLE) or systemic sclerosis (SSc), normal cognitive functions are essential for performing daily activities. These diseases may be associated with cognitive dysfunction (CD). In RA, CD has been associated with age, lower education and disease duration and activity. Great advances have been achieved in neuropsychiatric SLE in the identification of pathogenic pathways, assessment and possible treatment strategies. SSc rarely exerts direct effects on the brain and cognitive function. However, the psychological burden that includes depression, anxiety and social impact may be high. AIRD patients with sustained disease activity, organ damage or lower education should be evaluated for CD. The control of systemic inflammation together with tailored behavioural cognitive therapies may benefit these patients.

## Introduction

Cognitive function includes orientation, attention/concentration, judgment/problem solving and memory, verbal, visual/spatial and executive functions [1]. Several standardised measures of cognitive function have been developed. These include the Montreal Cognitive Assessment (MoCA), Trail Making Test (TMT), Victoria Stroop Test (VST), Wechsler Adult Intelligence Scale (WAIS) and Benton Visual Retention Test (BVRT). Beck Depression Inventory (BDI) and State-Trait Anxiety Inventory (STAIT/S) may be used to assess depression and anxiety, respectively [2]. For people with chronic illnesses, such as autoimmune-inflammatory rheumatic diseases (AIRD), intact cognitive functioning is crucial for performing many key daily tasks, including medical treatment adherence or planning activities. Most AIRDs have been associated with various degrees of cognitive dysfunction (CD) (reviewed in [1, 3, 4]). In this review, we will briefly discuss CD in rheumatoid arthritis (RA), systemic lupus erythematosus (SLE) and systemic sclerosis (SSc).

## Rheumatoid arthritis

### Introduction

RA exerts various neuropsychiatric manifestations [5]. There is an increased risk of stroke, a consequence of accelerated inflammatory atherosclerosis, especially in elderly RA patients with a long-standing disease [5]. Among psychiatric manifestations, depression and anxiety are present in two thirds of RA patients and have been associated with disease activity [6].

In RA, patients may significantly underperform on cognitive function tests compared to controls [1, 2, 7]. Even mild CD may influence the functional capacity and quality of life of these patients [1, 7, 8]. It affects reactivity to pain, compliance to and effectiveness of therapy [1]. This CD may occur early, in young RA patients, even in the early stages of the disease [9]. In general, CD may influence daily activities, treatment compliance and overall self-care [1, 2]. Meade et al. [1] have recently performed the first systematic review of CD in RA.

### Pathogenesis

Both inflammation itself that also affects the brain [1, 8] and accelerated atherosclerosis also driven by systemic inflammation including pro-inflammatory cytokines [10] may cause CD in RA. RA disease activity associated with systemic inflammation may be an important driver of CD, as well as depression and anxiety [1, 8]. Clinical features, such as pain, stress, fatigue and sleep disturbances, may be involved in the development of RA-associated CD. For example, depression can lead to abnormal concentration and executive function [1, 11]. Among anti-rheumatic drugs, methotrexate (MTX) and corticosteroids have been associated with CD. The effects of these compounds on RA-related CD may be controversial. Both MTX and corticosteroids dampen systemic inflammation and thus may have beneficial effects on cognitive function. On the other hand, MTX has been associated with CD, mood changes and confusion, while corticosteroids may have an impact on memory and hippocampal function [1, 12].

### Assessment tools

We have recently applied numerous standard validated measures in order to assess cognitive function, depression and anxiety in RA patients in association with the assessment of cerebral circulation and pathology by transcranial Doppler (TCD), carotid ultrasound and brain MRI [2]. Standard measures of CD including MoCA, TMT, VST, WAIS and BVRT, as well as BDI and STAI measuring depression and anxiety, showed impairment in RA vs controls. Biologic- vs MTX-treated subgroups also showed some difference in a few measures [2]. Shin et al. [7] used 16 cognitive indices and found a relationship between cognitive and functional limitations. We have reported the impairment of most mental domains in the SF-36 quality of life assessment tool in RA [2]. In our study, numerous cognitive tests correlated with age and lower education. Some also correlated with disease duration, ESR and disease activity [2]. Shin et al. [7] and Lee et al. [13] also associated CD with low education and disease activity, respectively. Regarding vascular pathophysiology, cerebral vascular lesions, TCD parameters and carotid plaques were associated with impairment of multiple cognitive parameters [2].

### Management

The control of disease activity may be essential in this respect [1, 8]. Although preliminary studies suggested that MTX may aggravate CD, the beneficial effects of MTX and biologics on the suppression of disease activity may override this potential effect [1]. Although pro-inflammatory mediators have been implicated in the development of CD in RA [1, 8], only very few studies evaluated the longitudinal effects of anti-rheumatic drugs on cognitive function. In the open-label study of Raftery et al. [14], adalimumab improved full scale, verbal and performance IQ in parallel with improvements of DAS28. Camara et al. [15] reported that centrally administered etanercept improved anxiety-like behaviour in mice. In addition to pharmacotherapy, tailored cognitive-behavioural therapy (CBT) administered early in the course of RA also reduced health care use for the first 5 years after treatment [16].

## Systemic lupus erythematosus

### Introduction

Neuropsychiatric manifestations in SLE, formerly known as lupus cerebritis, are diverse and common. An American College of Rheumatology (ACR) ad hoc committee in 1999 described 19 neuropsychiatric syndromes that can be directly attributed to SLE, ranging from stroke, acute confusional state and transverse myelitis to more low-grade, non-specific symptoms such as headaches, mood disorders and cognitive impairment [17]. While often considered subtle and non-specific, lupus patients have identified CD as one of the most distressing symptoms of their disease. Studies have estimated that CD affects 3–81% of lupus patients [3], the wide range resulting from non-standardised diagnostic criteria and screening tools, and difficulty in the attribution of symptoms to SLE. Additional hurdles in the diagnosis of neuropsychiatric manifestations are that they often develop insidiously, can present and progress independently of other signs of SLE activity and often do not respond to standard immunosuppression. Thus, there is growing recognition that CD in lupus patients remains underdiagnosed and under-addressed in the clinical setting [18].

### Pathogenesis

Suffering from a chronic disease state, taking neurotoxic/psychoactive medications such as corticosteroids and cyclophosphamide, and other manifestations of neuropsychiatric SLE (NPSLE) such as strokes, seizures, depression or anxiety can all independently lead to CD. Nevertheless, the early presentation of neuropsychiatric symptoms (40% of lupus patients suffer from neuropsychiatric symptoms on presentation or within the first 3 years of disease diagnosis [19, 20]), the increased prevalence of CD in lupus patients compared to other chronic inflammatory diseases [3], in addition to animal models displaying associated behaviours [21], strongly points to a disease-specific, immunologic aetiology. Pathogenic pathways thought to be instrumental in NPSLE have been comprehensively described elsewhere [21], but here, we will briefly discuss those that have been specifically linked to cognitive dysfunction. Antiphospholipid (aPL) antibodies, often associated with SLE and known to be a strong risk factor for NPSLE, lead to a hypercoagulable state often resulting in strokes, structural damage and associated CD [21, 22]. Of note, aPL antibodies have also been linked to less clearly understood NPSLE syndromes that are not necessarily directly related to thrombosis or ischaemic events [23]. In fact, there is evidence for the involvement of multiple aspects of the immune system in NPSLE, including neurotoxic autoantibodies, pro-inflammatory cytokines and cell-mediated effects, in conjunction with abnormalities in neuroimmune interfaces including the choroid plexus and blood-brain barrier which allow systemic autoimmune drivers into the central nervous system [21, 24]. Specifically, several studies have pointed to increased systemic levels of cytokines such as interleukin 6 (IL-6) and neurotoxic anti-*N*-methyl-d-aspartate receptor (NMDAR) antibodies in SLE patients with CD [25–27]. In addition, type I interferon (IFN-I) and anti-NMDAR antibodies were shown to enhance microglia activation, leading to aberrant synaptic pruning with subsequent CD [28, 29]. There is some experimental evidence to suggest that microglia activation can be dampened with both anti-IFN-α/β receptor (IFNAR) treatment and the blood-brain barrier-crossing angiotensin-converting enzyme (ACE) inhibitor captopril. These interventions were associated with improvement in neurobehavioural deficits in lupus mice [28, 29], although there was a conflicting report with anti-IFNAR treatment and its effect on neuropsychiatric symptoms [30].

While promising directions in terms of investigations into the pathogenesis, as well as specific treatments of CD, are starting to accrue, those are still mainly focused on animal disease models, limiting our ability to extrapolate these findings to human disease [21, 28–30].

### Measurement tools

The ACR 1999 nomenclature defined CD as including a significant deficit in any or all of the following cognitive domains: simple or complex attention, reasoning, executive skills, memory, visual-spatial processing, language and psychomotor speed [17]. Among these, in SLE, language and working memory domains are most commonly affected, followed by simple attention and psychomotor speed [3]. Rayes et al. [3] presented a comprehensive meta-analysis evaluating different neurocognitive testing tools used to assess CD in SLE. While the 4-h, complete battery of neurocognitive testing is the most comprehensive, it is not feasible for routine assessments of patients in the clinical setting. A number of alternative modalities have been utilised with varying degrees of agreements with the complete neurocognitive testing battery, including the ACR-SLE battery [17], a 1-h battery of tests that focus on SLE-specific domains that show good agreement with the complete battery in healthy controls and SLE patients without NPSLE (96% and 95% agreement, respectively, with 100% sensitivity in both groups, and 96% specificity in controls and 94% in SLE without NPSLE), but only moderate agreement in patients with NPSLE (81% agreement, with 80% sensitivity and 81% specificity) [31]. While the ACR-SLE battery has been shown to be a relatively good CD evaluating tool in SLE patients, its main barrier is the significant time and resources it requires for administration, making it challenging for routine use. The Automated Neuropsychological Assessment Metric (ANAM) is a 40-min, self-administered computerised test that has been validated in multi-ethnic populations and is less influenced by age, education, English language proficiency and depression than other forms of neurocognitive testing [32, 33]. When compared to the ACR-SLE battery, the ANAM was found to have a sensitivity of 78–80% and specificity of 70% [32, 33], demonstrating promise as a possible CD screening method. The ANAM, however, needs further analysis of its capacity to monitor changes in CD over time and is thought to lack the ability to identify specific domain impairments [3]. Finally, the MoCA test, already mentioned above, is a brief, 15-min administered test that showed acceptable agreement with the ANAM (sensitivity 83% and specificity 73%) but requires more extensive validation prior to clinical and investigational use [34]. Identifying and validating a reliable screening and monitoring tool for CD that is both sensitive and specific, as well as technically feasible in the clinical setting, are of the utmost importance. Relying on patients’ self-reporting is clearly inadequate, especially as studies comparing patient self-reporting to objective findings of CD found no association between the two, most probably because self-perception of CD is often closely associated with depression and anxiety [35]. It is also important to emphasise that these tests may be useful for the identification and quantification of CD, but are not specific for NPSLE, and do not allow the discerning of primary SLE-related CD from any other confounding causes.

### Advanced imaging studies

The pathways underlying lupus CD are still being explored, and the currently available diagnostic tools are not specific and/or have yet to be fully validated and broadly accepted. Thus, there remains a need for an objective and non-invasive means to establish neurological dysfunction in SLE patients both for investigative purposes, as well as for clinical diagnosis and surveillance. There is evidence to suggest that SLE patients, with and without overt NPSLE, have aberrations in their white matter microstructures as seen by diffusion tensor imaging (DTI) [36]. Functional MRI studies have shown increased functional connectivity in core resting-state networks in SLE patients that correlate with reduced cognitive performance compared with healthy controls [37]. FDG-PET imaging highlighted hypermetabolic regions that corresponded with increased serum anti-NMDAR antibodies in SLE patients with CD and did not correlate with disease duration, activity, medications or comorbidities [26, 27]. These studies highlight a potential role for these imaging techniques in objectively diagnosing and monitoring CD in SLE patients.

### Attribution of CD to SLE

The potential use of imaging studies to diagnose CD in SLE in an unbiased and disease-specific setting is appealing, especially as there are numerous confounders that can affect CD in SLE patients. As we noted, mood disorders, depression, chronic pain, corticosteroid use and struggling with a chronic illness from a young age are all known to contribute to CD and are quite common in SLE patients. In order to better study, address and manage CD symptoms in SLE patients, it is important to first be able to correctly attribute them to the disease itself. To that end, several models have been devised to aid clinicians in attributing the symptoms to SLE (comprehensively reviewed in [38]). These models take into account factors such as the temporal relationship between the onset of neuropsychiatric symptoms and diagnosis or change in the activity of SLE, viable alternative explanations and the presence of risk factors specific to SLE such as aPL antibodies [22]. However, these models have been developed with the expert opinion being the diagnostic gold standard, with no objective corroborating evidence. Therefore, the attribution of CD to SLE remains mainly reliant on the clinician’s assessment, index of suspicion and clinical reasoning, which can benefit from the introduction of a collaborative multidisciplinary approach including neuropsychological, radiological and laboratory evaluations [21, 39].

## Systemic sclerosis

### Introduction

Scleroderma encompasses several significant immune-mediated diseases that lead to thickening or fibrosis of the skin. The most severe forms medically are those in which systemic involvement occurs, namely SSc [40]. It has been suggested that the central nervous system is one of the very few organs that is not involved in SSc, but in fact, there are several aspects of the disease where cognitive function is clearly relevant and important. The localised forms of scleroderma, termed morphoea, are also important in terms of functional and cosmetic burden due to their impact on local connective tissue growth and appearance. Interestingly emotional stress is recognised as a factor in the development of scleroderma, and it is not uncommon for patients to describe major emotional difficulties such as bereavement or divorce in the 12–18 months prior to disease onset [41]. In this way, the interplay between the neurocognitive processes and immune or inflammatory disease is highlighted.

### Psychological burden in SSc

More specific issues occur due to the impact of SSc on physical appearance and function. This can impact on almost all aspects of daily life and has a major detrimental effect on social interaction. Reasons for this include the effect on appearance as well as difficulties related to eating, working or participating in normal sociable activities. The impact is clear from studies that have used validated instruments to assess burden [42]. In addition, there has been a clear demonstration that clinical interventions that can improve facial appearance, such as autologous fat transfer, can greatly improve the score of psychological well-being [43].

There have been recent attempts to better describe and quantify psychological burden in SSc, and these have used the randomised cohort design of the SPIN project [44]. This approach allows greater evidence gathering to assess the utility of measurement tools as well as possible intervention strategies [45]. This is important because the psychological impact of SSc is frequently highlighted by patients as a major unmet need and one that is not well addressed in current approaches to treatment [44, 45]. The shared aspects across other immune-mediated or chronic diseases may be important in planning future interventions.

### Cognitive impairment in SSc

In general, although anxiety, depression and mood difficulties are common, CD in SSc is not a frequent complication of the disease process. This contrasts with other AIRDs and might reflect fundamental differences in the cytokine or other immune cell drives across diseases [46]. There are however instances where vascular damage can be relevant. The most striking example is in the context of scleroderma renal crisis. This is a form of thrombotic microangiopathy associated with accelerated phase hypertension that occurs almost exclusively in early-stage diffuse cutaneous SSc and is strongly associated with the anti-RNA polymerase autoantibodies. There is a propensity to end-organ damage that may reflect the systemic microvasculopathy of SSc and may lead to generalised seizures and on occasion significant CD [47]. Fortunately, this generally recovers over several weeks without sequelae. A recent study formally assessed cognitive function in a cohort of SSc cases found evidence of cognitive impairment compared with matched healthy controls and that this associated with worse vascular disease. Future studies may explore whether this relates to comorbidity or is a specific feature of SSc [48].

Approximately one in five patients with SSc will also manifest additional features of another AIRD, such as SLE or Sjögren’s syndrome [49]. This is important because the cognitive impact of these other diseases will be an additional burden for patients and may sometimes be the predominant problem, as outlined in other parts of this article. Immune-mediated encephalitic states may occur occasionally in this context [50].

Finally, it is notable that SSc is associated with Raynaud’s phenomenon, and there have been several reports of mental or cognitive impact of cold exposure [4]. This has in some cases been associated with changes in cerebral perfusion scan and raises the intriguing possibility that Raynaud’s might on occasion affect the cerebral circulation [4]. This has not been definitely shown, and more research is needed. Likewise, in localised scleroderma, especially linear morphoea, there can be structural abnormalities affecting the cranium or brain substance. This has been associated with epilepsy and possibly also with cognitive complications in some cases.

## Conclusion

Patients with RA, SLE or SSc may exert significant cognitive impairment. In RA, various domains of cognitive dysfunction may be associated with age, lower education, disease duration and activity, inflammatory markers and some measures of vascular pathology. Important strides have been made in NPSLE- and SLE-specific CD in particular, in the elucidation of the pathogenic pathways and possible treatment strategies, imaging studies, and the design and validation of clinically feasible and diagnostically acceptable screening and monitoring tools. SSc rarely directly affects brain function, but the psychological burden can be large including depression anxiety and social impact. This represents a major unmet need that will likely remain a key problem despite promising data from ongoing and recent clinical trials of potential therapies for skin or lung manifestations of the disease. Although there have been fewer studies of cognitive impairment in SSc that for SLE or RA, it is important and maybe addressed in future work, perhaps utilising the randomised cohort design of the SPIN consortium [44]. From the practical point of view, AIRD patients, especially those with higher age, lower education, sustained disease activity and higher damage indices, should be evaluated for CD by any standard test. Although some anti-rheumatic agents may impair cognitive function, the control of inflammation by conventional and biologic DMARDs, in association with tailored behavioural cognitive therapies, may benefit our AIRD patients. There obviously is still much work to be done in developing the right tools to best serve and optimally manage these patients with this distressing complication of an already challenging disease.

## Data Availability

This is a review. There are no data in this manuscript; therefore, this section is not applicable.
